# Late Miocene–Pliocene Paleoclimatic Evolution Documented by Terrestrial Mollusk Populations in the Western Chinese Loess Plateau

**DOI:** 10.1371/journal.pone.0095754

**Published:** 2014-04-21

**Authors:** Fengjiang Li, Naiqin Wu, Denis-Didier Rousseau, Yajie Dong, Dan Zhang, Yunpeng Pei

**Affiliations:** 1 Key Laboratory of Cenozoic Geology and Environment, Institute of Geology and Geophysics, Chinese Academy of Sciences, Beijing, China; 2 Laboratoire de Meteorologie Dynamique, UMR INSU-CNRS 8539 & CERES-ERTI, Ecole Normale Superieure, Paris, France; 3 Lamont-Doherty Earth Observatory of Columbia University, Palisades, New York, United States of America; 4 School of the Earth Sciences and Resources, China University of Geosciences, Beijing, China; University of Florence, Italy

## Abstract

The Neogene eolian deposits in the Chinese Loess Plateau (CLP) are one of the most useful continental deposits for understanding climatic changes. To decipher Late Neogene paleoclimatic changes in the CLP, we present a terrestrial mollusk record spanning the time interval between 7.1 and 3.5 Ma from the western CLP. The results indicate four stages of paleoclimatic evolution: From 7.1 to 6.2 Ma, cold and dry climatic conditions prevailed as evidenced by high values of the total number of cold-aridiphilous (CA) mollusk species and by low values of all of the thermo-humidiphilous (TH) mollusk indices. From 6.2 to 5.4 Ma, the climate remained cold and dry but was not quite as dry as during the preceding phase, as indicated by the dominance of CA mollusks and more TH species and individuals. From 5.4 to 4.4 Ma, a warm and moist climate prevailed, as indicated by high values of the TH species and individuals and by the sparsity of CA species and individuals. From 4.4 to 3.5 Ma, all of the CA indices increased significantly and maintained high values; all of the TH indices exhibit high values from 4.4 to 4.0 Ma, an abrupt decrease from 4.0 Ma and a further increase from 3.7 Ma. The CA species of *Cathaica pulveraticula*, *Cathaica schensiensis*, and *Pupopsis retrodens* are only identified in this stage, indicating that the CA species were diversified and that the climate was becoming drier. Moreover, the CA mollusk group exhibits considerable diversity from 7.1 to 5.4 Ma when a cold, dry climate prevailed; whereas the diversity of the TH group was high during the relatively warm, wet interval from 5.4 to 4.4 Ma. This indicates that variations in the diversity of the CA and TH mollusk groups were closely related to climatic changes during the Late Miocene to Pliocene.

## Introduction

As evidenced by the marine benthic foraminiferal δ^18^O record, Earth’s climate underwent a gradual cooling trend during the Late Miocene and Pliocene. This interval witnessed the progressive cooling of oceanic deep water, the expansion of permanent ice sheets in Antarctica, the occurrence of ice rafted detritus in Northern Hemisphere (mostly in north Atlantic), and both hemispheres covered by ice sheets after middle Pliocene [1]. Coincident with ice development in both Polar regions are significant ecological, climatic, and tectonic events elsewhere and especially around Asia [1]–[12], and which demonstrate that the Late Miocene and Pliocene was an important and complex interval that needs to be better understood.

**Figure 1:**
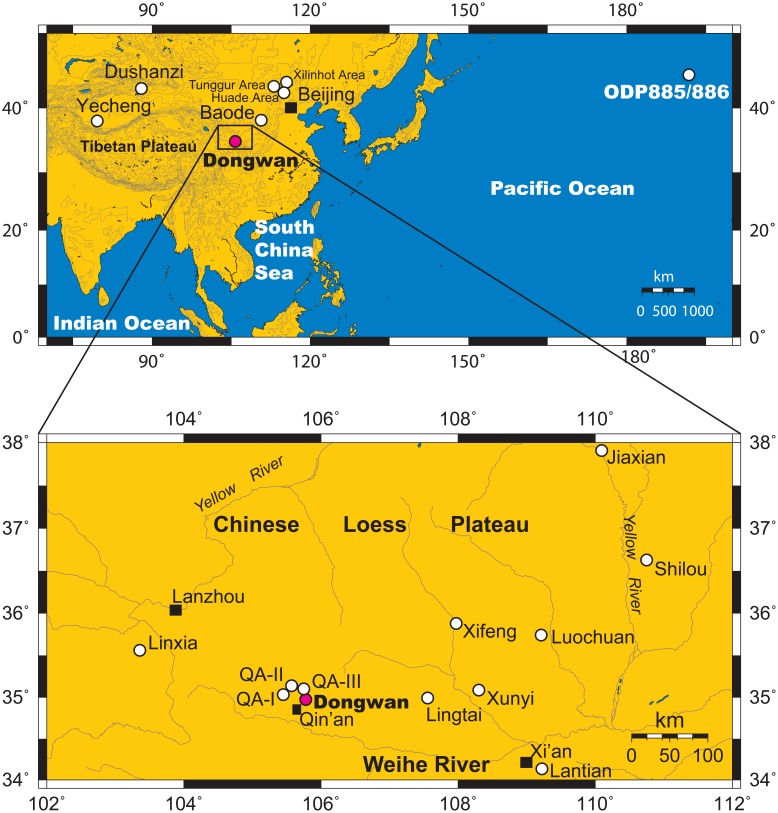
Location of the Chinese Loess Plateau (CLP), the studied Dongwan loess-paleosol sequence and other Neogene sections mentioned in the text. The Dongwan sequence is indicated by a red circle and other sections by white circles. Main cities are indicated by black squares.

In East Asia, one of the most important climatic changes is the evolution of the East Asian (EA) monsoon. Numerous sedimentological, geochemical, and paleontological studies, including of fossil mammals, mainly from the Chinese Loess Plateau (CLP) and the South China Sea (SCS), have contributed significantly to our understanding of the EA monsoon changes during the Late Miocene to Pliocene. However, the results are inconsistent and some are even in conflict. In the CLP, sedimentological evidence of changes in sediment grain size and sedimentation rate from the Xifeng, Lingtai, Lantian, Xunyi, Luochuan, Jiaxian, Baode, and Jingle (Figure 1) Red Clay deposits indicate the occurrence of a strong winter monsoon and pronounced aridity in the Asian interior during the Late Miocene [8], [13]–[16]. However, the geochemical record of ^87^Sr/^86^Sr ratios from the Lingtai Red Clay sequence implies a weak EA winter monsoon from 7 to 2.5 Ma [17]; and in addition, this interval can be sub-divided into a large amplitude and high frequency stage from 7–4.2 Ma and a weak, stable stage from 4.2–2.6 Ma, as evidenced by records of Zr/Rb ratio and mean quartz grain size [18]. Furthermore, grain-size records from fluvial deposits in the Linxia Basin (Figure 1) indicate that the EA winter monsoon intensified at 7.4 Ma and 5.3 Ma [19], the latter datum being significantly different to evidence from the Red Clay record.

In the case of the EA summer monsoon, the magnetic susceptibility record from the Xifeng Red Clay sequence indicates a weakened summer monsoon during the Late Miocene (6–5.4 Ma) and a strong summer monsoon during the early Pliocene [8]. Additional information about the EA summer monsoon is provided by records of pedostratigraphy and iron geochemistry from the Lingtai Red Clay deposits. These records indicate that the EA summer monsoon was relatively weak from 7.05 to 6.2 Ma, strengthened from 6.2 to 5.5 Ma, was very strong from 5.5 to 3.85 Ma corresponding to the interval of strongest soil development, and weakened significantly over the interval 3.85 to 3.15 Ma [20], [21].

However, studies of mammalian fossils from the CLP have yielded very different results: Hypsodonty analysis indicates that northern China became more humid at 7–8 Ma, coincident with the onset of Red Clay deposition in the eastern CLP, and which was interpreted by the authors as representing the onset or intensification of summer monsoonal precipitation [22], [23]. A general rise in δ^18^O values of soil carbonate from the Lantian fluvial and Red Clay deposits reflects increased summer precipitation related to the onset and/or intensification of the EA monsoon during the Late Miocene to Pliocene [24], [25], and which is supported by a mammalian faunal turnover event implying a marked change to more humid and closed habitats [26]. Recent isotopic evidence from fossil mammals and soil carbonates indicates a strengthened EA summer monsoon from 7–4 Ma [27]. However, the δ^13^C values of fossil enamel from a diverse group of herbivores and of paleosol carbonate and organic matter from the Linxia Basin indicate that C4 grasses were either absent or insignificant in the Linxia Basin prior to 2–3 Ma, suggesting that the EA summer monsoon was probably not strong enough to affect this part of China throughout much of the Neogene [28]. However, this result conflicts with the interpretations of Fortelius et al. (2002) [22], Kaakinen et al. (2006, 2013) [24], [25], Liu et al. (2009) [23] and Passey et al. (2009) [27].

In the SCS, the EA winter monsoon developed progressively from 7 Ma as shown by an increasing trend in black carbon concentration and accumulation rate [29]. In addition the EA summer monsoon weakened after 7.5 Ma, as evidenced by combined planktonic foraminiferal Mg/Ca and δ^18^O records [30], consistent with the Red Clay sequences in the CLP. The suggestion of a weakened EA summer monsoon is supported by geochemical records which indicate that chemical weathering intensity decreased from the early Miocene with a rapid decrease centered at 7.2 Ma [31]. This result is generally consistent with records of radiolarian species numbers and individuals, and diversity, from the southern SCS and which suggest a major decrease in summer monsoon intensity after 7.70 Ma [32]. In contrast, records of clay/feldspar ratio, kaolinite/chlorite ratio and biogenic opal MAR from the SCS suggest that the summer monsoon was strong from 7.1–6.2 Ma and remained relatively stable from 6.2–3.5 Ma [33]; however, the authors emphasize that their study is a schematic reconstruction which only outlines the principal stages but neglects the details.

A semi-quantitative reconstruction of the Neogene vegetation in China indicates that Miocene aridification associated with strengthening of the EA winter monsoon is consistent with Neogene global cooling, and that the EA summer monsoon did not weaken during the Pliocene [34], and this result agrees with most of the geological records from the CLP and SCS. However another quantitative reconstruction from plant fossil records yielded contrasting results, indicating that records of both temperature and precipitation from north China exhibit no significant difference between the western and eastern regions during the Miocene, suggesting that the monsoon climate did not commence or intensify at that time [35]. Regional climate model experiments also reveal that during the Late Miocene, from 11–7 Ma, the monsoonal climate may not have been fully established in various Asian regions, including northern China [36]; and this finding is contrary to that of numerous previous studies of the EA monsoon which suggest that it was initiated around the time of the Oligocene/Miocene boundary [37]–[39].

The foregoing review demonstrates that more work is needed to better understand the evolution of the EA monsoon during the Late Miocene and Pliocene. In particular, higher resolution studies using more sensitive monsoon proxies from key monsoon-dominated regions may be one of the best solutions for resolving the various inconsistencies and even conflicts regarding the process of monsoon development.

As mentioned above, the CLP (Figure 1), located to the northeast of the Tibetan Plateau, is a key continental region for the study of the EA monsoon. Deposition of eolian sediments commenced in the western CLP from 22 Ma, as observed in the QA-I, QA-II, and QA-III (Figure 1) Miocene loess-paleosol sequences which have a basal age of about 22 Ma [37], [40], while the upper boundary is dated at about 3.5 Ma in the Dongwan late Miocene-Pliocene loess-paleosol sequence in the western CLP [41]. In contrast, in the eastern CLP, the age of the lower boundary of eolian deposits, the Red Clay sequences, is about 7–8 Ma as evidenced in the Lingtai, Xifeng, Liantian, and Baode sequences [15], [20], [42], [43]. However, results from the recently reported Shilou Red Clay sequence indicate that eolian sediments were deposited from 11 Ma in the eastern CLP [44]. Despite the occurrence of a totally different lower boundary age between the east and west CLP, these deposits have great potential for deciphering in detail the processes of ecological and climatic evolution in the CLP, and by extension in East Asia, during the Miocene and Pliocene. As reviewed above, sedimentological and geochemical studies have so far contributed a great deal to our understanding of climatic changes during the interval of interest [6], [8], [9], [13]–[18], [20], [21], [24], [27], [37], [40]–[45]; however, to date there has been only a limited application of a biological approach to analyzing these sequences [22], [26], [46], [47].

Land snails are generally the most common and abundant fossils in Quaternary loess sequences, and this fact, together with the limited degree of success of most of the other paleontological studies of Quaternary loess, makes them especially important paleoenvionmental indicators for loess deposits. Their occurrence in Quaternary loess was first documented in Europe in the early 1820s [48], and since then they have contributed significantly to understanding the origin and paleoclimatic evolution of Quaternary loess deposits in Eurasia and especially in the CLP [46], [49]–[57]. However, terrestrial mollusks preserved in the Neogene sequences in the CLP have not been investigated in detail until recently. Although they have provided crucial paleontological evidence for the wind-blown origin of the Neogene loess sequences in the western CLP [58], [59], the ecological and climatic information that terrestrial mollusks may provide has not been well deciphered, with the exception of the record from the Xifeng Red Clay sequence spanning the interval from 6.2 to 2.4 Ma in the eastern CLP [46]. Furthermore, it is unknown how terrestrial mollusk diversity varied in the CLP against the background of climate changes during the Neogene, since the necessary studies have not been performed. In this study, we studied the record of terrestrial mollusks preserved in the Late Miocene to Pliocene Dongwan section in order to investigate the evolution of paleoclimate and terrestrial mollusk diversity in the western CLP during the Late Miocene to Pliocene.

## Materials and Methods

The Dongwan loess-paleosol sequence (105°47′E, 34°58′N) [41] is located in the northeastern part of Qin’an County in the western CLP (Figure 1). The current climate in Qin’an County is mainly controlled by the EA monsoon, with mean annual precipitation of about 400–500 mm and mean annual temperature of ∼10.4°C. Mean July and January temperatures are ∼22.7°C and −3.4°C, respectively. Vegetation in the region corresponds to a semi-arid temperate steppe [60].

**Figure 2:**
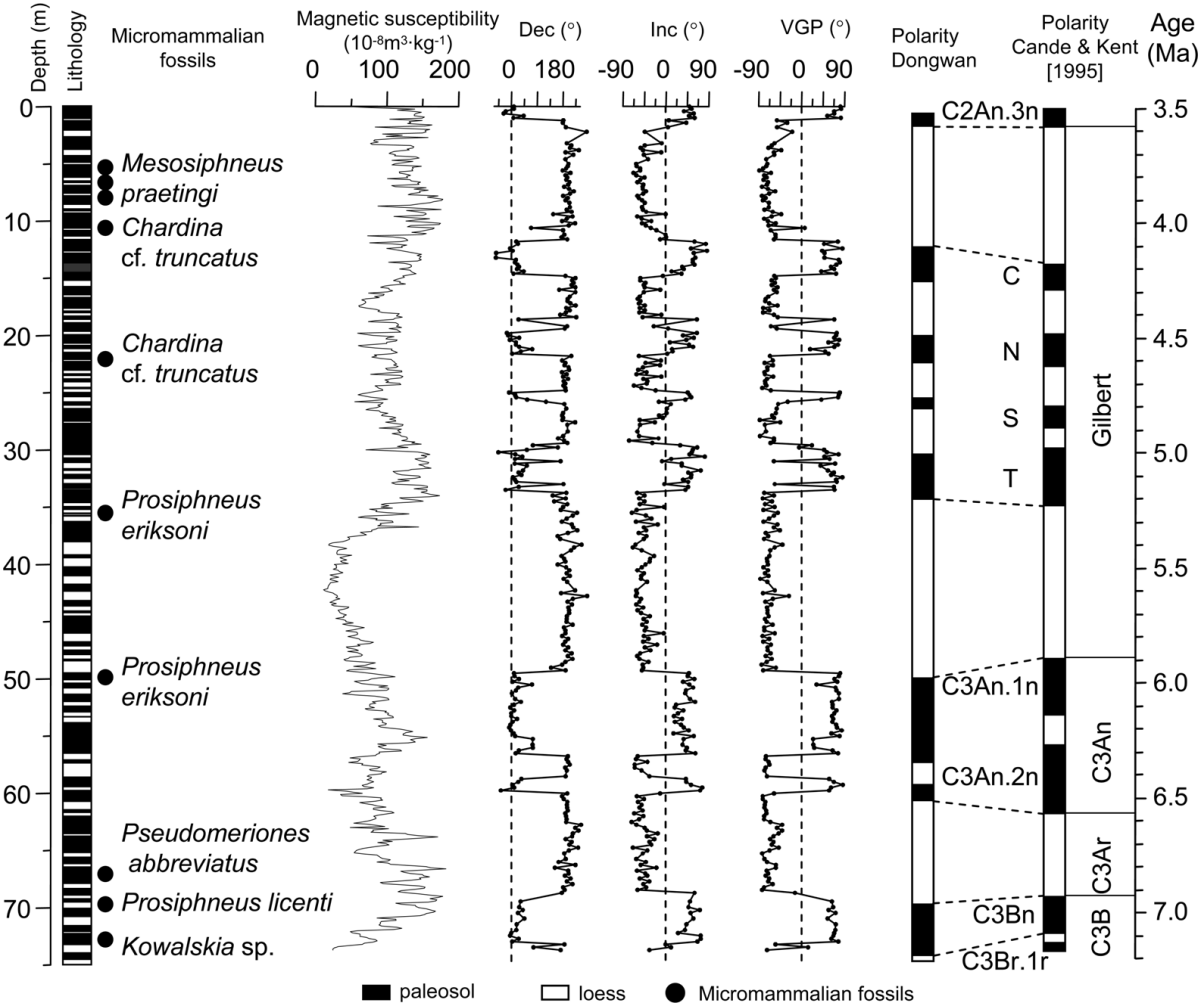
Chronology of the Dongwan loess-paleosol sequence (modified after Hao and Guo, 2004) [41].

The Dongwan sequence, located in the western CLP, is the first counterpart of the Red Clay sequences in the eastern CLP [41]. The section is about 73.7 m thick, and is composed of 84 distinguishable loess-paleosol couplets. The chronology of the sequence has been established by Hao and Guo (2004) [41] using magnetostratigraphic and micromammalian studies (Figure 2). First, Hao and Guo (2004) [41] ascribed the approximate age of the sequence to the Late Miocene to Pliocene using micromammalian fossil teeth sampled from 20 depths of the Dongwan sequence. Second, they defined a series of magnetozones based on 319 oriented samples collected at 20 or 25 cm intervals and correlated them with the Geomagnetic Polarity Timescale [61]. Finally, they established a chronology using paleomagnetic reversals as age controls and interpolated in between them using the magnetic susceptibility age model developed by Kukla et al. (1990) [62]. Their results yield an age duration of 7.1 to 3.5 Ma for the Dongwan loess-palaeosol sequence [41].

A total of 310 mollusk assemblages were collected from the Dongwan sequence using continuous 20-cm-thick samples; however, some intervals were sampled at intervals of 10–50 cm, according to lithological changes [58]. About 30 kg of sediment were obtained for each sample. In the field, we progressively broke each sediment sample into smaller pieces of about 0.5 mm in diameter, at the same time collecting all available shells and visible broken pieces. No necessary permits for the described field investigations were needed. In the laboratory, we attempted to restore any broken shells, and then identified and counted them under a binocular microscope. All of the identifiable mollusk remains were considered in the individual totals using the method of Puisségur (1976) [63]. All of the mollusk shells are stored in the Institute of Geology and Geophysics, Chinese Academy of Sciences.

For each mollusk assemblage, the numbers of species (S) and individuals were counted and a diversity index was calculated for all species, thermo-humidiphilous (TH) species, and cold-aridiphilous (CA) species in order to investigate changes in terrestrial mollusk populations and in different ecological groups. We used the most widely applied Shannon index [64], sometimes referred to as the ‘Shannon–Weaver’ index and sometimes as the ‘Shannon–Wiener’ index by researchers, in order to calculate the values of diversity, *H* (S), of the total, TH, and CA species, as follows:
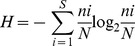
where *n_i_* is the density measure (in this case the mollusk individuals) of the *i*-th species (*I* varying between 1 and *n*); *S* is the number of species in the sample, and



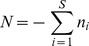



The theoretical maximum (*H*_max_) of diversity in any sample is expressed as
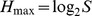



Equitability (or evenness, E) is expressed as
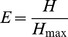



These indices have been applied to European and North American Quaternary terrestrial mollusk assemblages [65]–[67].

## Results

**Figure 3:**
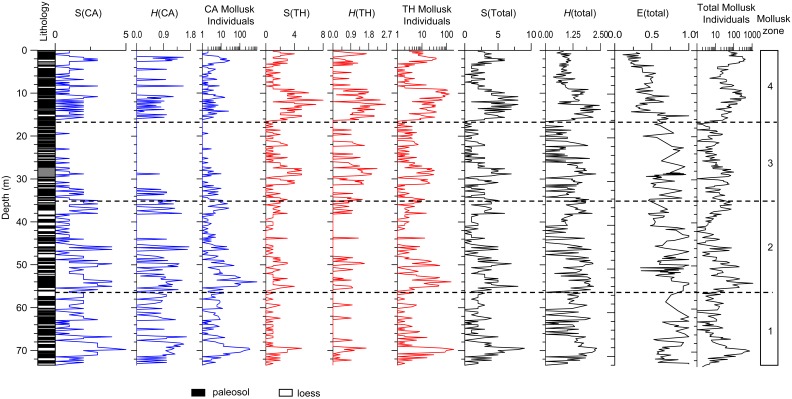
Variations in terrestrial mollusks versus depth in the Dongwan sequence. Lithology and total mollusk individuals are from Li et al., (2006a) [58] and Li et al. (2008) [47]. S(CA)–Total number of species of the cold-aridiphilous (CA) mollusk group. *H*(CA)–Diversity of the CA mollusk group. S(TH)–Total number of species of the thermo-humidiphilous (TH) mollusk group. *H*(TH)–Diversity of the TH mollusk group. S(total)–Total number of species of the total mollusk group. *H*(total)–Diversity of the total mollusk group. E(total)–Equitability of the total mollusk group.

Mollusk fossils are relatively abundant in the Dongwan sequence with significant concentrations at ∼2 m, 10 m, 30 m, 50 m, and 70 m depth. Amongst the total of 310 samples, 298 yielded 16439 mollusk individuals and 12 samples were barren. The maximum count reached 1121/30 kg at 54 m depth (Figure 3). Variations in total mollusk individuals parallel fluctuations in magnetic susceptibility [58], indicating that pedogenic processes such as carbonate dissolution did not affect the preserved assemblages [58].

A total of 24 mollusk species were identified in the Dongwan section. They are all terrestrial taxa and consist of *Gastrocopta armigerella* (Reinhardt, 1877), *Gastrocopta* sp., *Punctum orphana* (Heude, 1882), *Punctum* sp., *Metodontia huaiensis* (Crosse, 1882), *Metodontia yantaiensis* (Crosse et Debeaux, 1863), *Metodontia beresowskii* (Moellendorff, 1899), *Metodontia* cf. *huaiensis*, *Metodontia* cf. *yantaiensis*, *Metodontia* cf. *beresowskii*, *Metodontia* sp., *Kaliella* sp., *Macrochlamys* sp., *Opeas* sp., *Cathaica* sp., *Cathaica pulveratrix* (Martens, 1882), *Cathaica pulveraticula* (Martens, 1882), *Cathaica schensiensis* (Hilber, 1883), *Cathaica placenta* (Ping et Yen, 1933), *Pupilla aeoli* (Hilber, 1883), *Pupilla grabaui* (Ping, 1929), *Pupilla* sp., *Vallonia* sp., and *Pupopsis retrodens* (Martens, 1879).

All of these species, except *Pupilla grabaui* and *Pupopsis retrodens,* have been previously identified in the Chinese Quaternary loess-paleosol sequences and most of them have modern representatives. For example, *Cathaica pulveratrix*, *C. pulveraticula*, *C. schensiensis*, and *Pupilla aeoli* are the most common species that prefer living in relatively cold, dry environments, and are presently distributed in northwestern China. They have been regarded as indicative of a strengthened winter monsoon [49]–[58]. Conversely, *Metodontia huaiensis, M. yantaiensis*, *M. beresowskii, Gastrocopta armigerella,* and *Punctum orphana* are species living in a warmer and more humid environment, and are distributed mainly in the southeastern part of the CLP, where the warm, moist summer monsoon brings sufficient precipitation [49]–[58]. Therefore based on their present requirements of moisture and temperature, as well as their modern geographical distribution, the Dongwan terrestrial mollusks can be grouped into CA and TH ecological groups, as have been previously defined in the Chinese Quaternary loess-paleosol sequences [49]–[57]. In the Dongwan section, the TH group comprises *Gastrocopta armigerella*, *Gastrocopta* sp., *Punctum orphana*, *Punctum* sp., *Metodontia beresowskii*, *Metodontia huaiensis*, *Metodontia yantaiensis*, *Metodontia* cf. *huaiensis*, *Metodontia* cf. *yantaiensis*, *Metodontia* cf. *beresowskii*, *Metodontia* sp., *Kaliella* sp., *Macrochlamys* sp., and *Opeas* sp.; and the CA group comprises *Cathaica* sp., *Cathaica pulveratrix*, *Cathaica pulveraticula*, *Cathaica schensiensis*, *Cathaica placenta*, *Pupilla aeoli*, *Pupilla grabaui*, *Pupilla* sp., *Vallonia* cf. *pulchella*, *Vallonia* sp., and *Pupopsis retrodens*.

The distributions of these terrestrial mollusks in the Dongwan section have been described previously [58]. *Cathaica* sp. is the most continuously distributed mollusk taxon in the Dongwan section, and *Gastrocopta* sp., *Pupilla* sp., and *Vallonia* sp. are other well represented taxa. The TH species of *Metodontia*, *Punctum*, *Macrochlamys*, and *Opeas* are concentrated in the upper part of the section. In general, the CA species are dominant in the loess layers, while the TH species mainly occur in the paleosols [58].

Variations in the number of species (S), diversity (*H*), equitability (E), and total individuals of all species, CA species, and TH species in the Dongwan sequence are plotted against depth in Figure 3. These data can be found in the supporting information data (Table S1). Variations in these parameters allow definition of four mollusk zones. In Zone 1, from the bottom of the sequence to about 56.8 m depth (∼7.1–6.2 Ma), high values of all the CA species (S(CA)) and individuals and diversity (*H*(CA)) dominate over low values of all of the TH species (S(TH)) and individuals and diversity (*H*(TH)). A prominent feature of this zone is that high values of S(CA), *H*(CA), CA mollusk individuals, S(TH) and *H*(TH), as well as total mollusk species and individuals, occur at around 70 m depth, at the very base of the sequence; however, these values then decrease upwards. *H*(CA) and *H*(total) remain at high levels throughout the zone, and the equitability (E) of total species is also higher than in the other zones.

In Zone 2, from 56.8–34.8 m depth (∼6.2–5.4 Ma), the total number of CA species and individuals remains at a similarly high level as in the previous zone, except for the interval from 42 to 38 m. In contrast, the total number of TH species and individuals is slightly higher than in the previous zone. The total number of species and individuals of all of the species (CA and TH combined) are generally higher than in the previous zone but with a decreasing trend. The diversity of all species, *H*(total), does not exhibit significant changes compared to Zone 1, and E(total) is somewhat lower than in Zone 1.

Zone 3, from 34.8–16.2 m depth (∼5.4–4.4 Ma), is characterized by low numbers of CA species and individuals being almost absent in the upper part, from 24 m to 16.2 m. This pattern of variation is paralleled by a clear increase of all the TH indices, including diversity. The total number of species and individuals of all of the species is lower than in Zone 2, except for the interval from about 32 to 28 m. There are no large magnitude changes in *H*(total) and E(total) within the zone.

Zone 4 corresponds to the depth from 16.2 m to the top of the sequence (∼4.4 to 3.5 Ma). The number of CA species and individuals increases markedly and maintains relatively high values throughout the zone. The TH indices exhibit high values from about 16.2 to 9 m and then decrease significantly to very low values up to 4 m, and increase again above about 4 m depth. All of the indices for all species, i.e., S(total), *H*(total), and E(total), increase markedly; however, *H*(total) and E(total) exhibit a generally declining trend, implying that the diversity of the terrestrial mollusk populations decreased and that the distribution of individuals of different species was uneven. The total mollusk individuals in this zone is high and remains relatively stable except for the interval between 8 and 4 m, a pattern which differs from the other three zones where the total number of mollusk individuals is high at the beginning of the zone and then declines thereafter.

Moreover, the variation in diversity of the CA and TH ecological groups from the entire Dongwan sequence, as exhibited in Figure 3, can be differentiated into two major intervals based solely on the Shannon index of the CA and TH groups; and this result probably reflects different ecological population dynamics. First, from the base of the Dongwan sequence to 34.8 m depth, the CA group is dominant, exhibiting high diversity with *H* values varying between 0 and 1.75 (mean of 0.52). In contrast, the diversity of the TH populations exhibits significantly low values, ranging from 0 to 1.58 (mean of 0.18) within this depth interval, although the diversity of all mollusk species, i.e. the sum of CA and TH, does not exhibit any clear changes. Second, from 34.8 m depth to the top of the sequence, the diversity of the CA group declines significantly with *H* values varying between 0 and 1.56 (mean of 0.14); and in contrast the TH group becomes dominant in terms of diversity with *H* values ranging from 0 to 2.64 (mean of 0.64). The diversity and equitability of all mollusk species also exhibit a significant change at about 16.2 m depth within this interval.

## Discussion

### Late Miocene and Pliocene Paleoclimatic Evolution in the Western CLP

**Figure 4:**
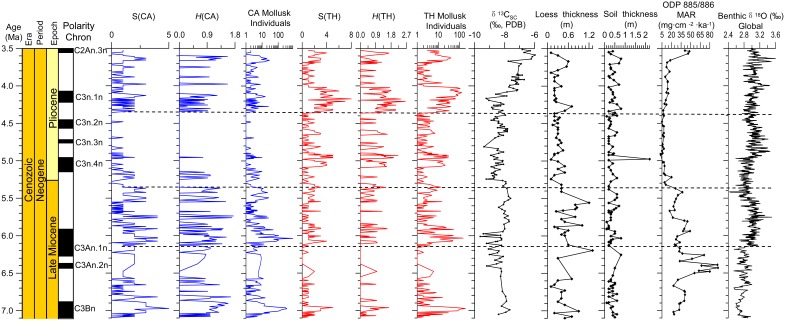
Variations in the terrestrial mollusks of the Dongwan sequence in the western CLP from 7.1 to 3.5 Ma, and comparison with other climate proxies. The proxies are δ^13^C of soil carbonate (δ^13^C_SC_) in the Lingtai Red Clay sequence [6], loess and soil thickness in the Dongwan sequence (this study), global cooling trend deduced from the marine benthic foraminiferal δ^18^O record [1], and continental aridity inferred from the mass accumulation rate (MAR) of eolian dust at ODP Site 885/886 in the western Pacific [69]. Geomagnetic polarity chronology and age of the Dongwan sequence is from Hao and Guo (2004) [41]. S(CA)–Total species of the cold-aridiphilous (CA) mollusk group. *H*(CA)–Diversity of the CA mollusk group. S(TH)–Total number of species of the thermo-humidiphilous (TH) mollusk group. *H*(TH)–Diversity of the TH mollusk group.

Previous studies of European and North American Quaternary terrestrial mollusks have shown that mollusk assemblages, based not only on the occurrence of characteristic species but also on the statistical dispersion of the assemblages (e.g., diversity, as described by the Shannon index (*H*)), can provide information about climatic conditions and mollusk populations [65]–[67]. For example, correlation of terrestrial mollusk groups with changes in dust flux, and thus climatic conditions, has previously been observed in the European Quaternary terrestrial mollusk diversity record [65]. Here we use the Dongwan terrestrial mollusk record to extend the statistical analysis of terrestrial mollusk populations and the interpretation of climatic changes to the Late Neogene. As shown in Figure 4, four stages can be recognized according to changes in the mollusk indices used in the present study, and which outline the evolutionary history of ecological and climatic conditions in the CLP from 7.1 to 3.5 Ma.

First, from 7.1 to 6.2 Ma (Zone 1), the total number of CA species (S(CA)) and diversity (*H*(CA)) index are high; however, the number of CA mollusk individuals is generally low apart from a large high peak at around 7 Ma and which indicates diversified CA species. Conversely, all of the indices of the TH species exhibit low values except for the peak at around 7 Ma, indicating low diversity. These features may be related to the occurrence of very cold, dry climatic conditions, the occurrence of which is roughly coincident with the expansion of C4 vegetation in the northeastern CLP [27], Central Inner Mongolia (Tunggur Area, Xilinhot Area, and Huade Area) [68] (Figure 1), and Pakistan [2]. They also indicate the occurrence of seasons with water stress for vegetation and terrestrial mollusks as well as the fact that relatively dry climatic conditions had already appeared in northern China during the Late Miocene, coincident in age with the global transition from C3 to C4 vegetation [4]. The occurrence of C4 plants during the latest Miocene should have been restricted to limited areas within deserts, and the occurrence of these niches could be coincident with, or occurred after, major uplift of the Tibetan Plateau at about 8 Ma [3], [8], [10], [11]. However, C4 vegetation was not yet to develop in the central and southern CLP, demonstrating that C3 plants were still dominant as indicated by δ^13^C values of soil carbonate in the Lingtai, Xifeng and Liantian Red Clay sequences in the eastern CLP [6], [9], [24]. This is also evidenced by the thickness of loess layers in the studied Dongwan sequence and by dust mass accumulation rates in the western Pacific. High dust deposition rates with a gradually increasing trend culminated at about 6.2 Ma in the western Pacific [69], corresponding to thicker loess layers in the Dongwan sequence (Figure 4). Both features indicate drier climatic conditions in the Asian interior, the potential source region of dust deposits in the CLP and western Pacific. While dust accumulation in the western Pacific reached a maximum from about 6.5 to 6.2 Ma, the number of mollusk species and individuals at Dongwan was very low, implying very dry climatic conditions which were unsuitable even for the development of CA species. Pedostratigraphy and iron geochemistry of the Lingtai Red Clay deposits in the CLP indicate that the EA summer monsoon was relatively weak from 7.05 to 6.2 Ma [20], [21]. The δ^13^C values of fossil enamel from a diverse group of herbivores and of paleosol carbonate and organic matter from the Linxia Basin indicate that C4 grasses were either absent or insignificant in the Linxia Basin prior to ∼2–3 Ma, suggesting that the East Asian summer monsoon was probably not strong enough to affect this part of China throughout much of the Neogene [28].

However, during our studied interval there is much evidence for a strong summer monsoon, evidenced mainly from mammal assemblages as well as analyses of mammal tooth and soil carbonate isotopes [22]–[25], [27]. Hypsodonty analysis of fossil mammals indicates that northern China became more humid at 7–8 Ma, coincident with the previously recognized onset of Red Clay deposition in the eastern CLP, and which was interpreted by the authors as reflecting the onset or intensification of summer monsoonal precipitation [22], [23]. Soil carbonate isotopes from the Lantian fluvial and Red Clay deposits indicate the absence of any marked change in plant photosynthetic pathway or climate, implying the occurrence of pure C3 vegetation with no indications of any C4 plants during the Late Miocene and Pliocene. A general rise in δ^18^O values probably reflects increased summer precipitation related to the onset and/or intensification of the Asian monsoon system [24], [25], which is supported by a mammalian faunal turnover event implying a marked change to more humid and closed habitats [26]. A strong summer monsoon at 7.1–6.2 Ma is also supported by the records of clay/feldspar ratio, kaolinite/chlorite ratio and biogenic opal mass accumulation rates (MAR) from the SCS, although the authors emphasize that their study is a schematic reconstruction which only outlines several principal stages and neglects the details [33].

There is also much evidence for a weakening of the summer monsoon and strengthening of the winter monsoon during the Late Miocene. The EA summer monsoon weakened after 7.5 Ma as evidenced by combined planktonic foraminifera Mg/Ca and δ^18^O records from Ocean Drilling Program (ODP) Site 1146, northern SCS [30]. Radiolarian species numbers and individuals and diversity from ODP Site 1143, southern SCS, suggest a summer monsoon maximum at 8.24 Ma and a major decline after 7.70 Ma [32]. Geochemical records from the ODP Site 1148, northern SCS, indicate that chemical weathering intensity decreased after the early Miocene with a rapid decrease centering at 7.2 Ma, compatible with a weakening of the EA summer monsoon [31]. The trend of increasing black carbon concentration and accumulation rate at ODP Site 1148 suggests the progressive development of the East Asian winter monsoon after about 7 Ma [29]. In addition, grain-size evidence from the Linxia Basin of the CLP indicates that the EA winter monsoon intensified after 7.4 Ma and again at 5.3 Ma [19].

Thus it is noteworthy that the TH mollusks at Dongwan do exhibit peak values at around 7 Ma, suggesting warm, humid climatic conditions consistent with a mammalian faunal turnover event [26]; however, the peak does not extend to the subsequent period, implying that the interval of relatively warm and humid climate may have been a relatively brief event within the context of an overall very cold and arid climate. In addition, a recent synthesis of isotopic and sedimentological analyses, climate modeling and an extensive mesowear analysis of the Baode Red Clay sequence indicates that the climate at 7.5 Ma was more humid than during the youngest interval at 5.7 Ma and that variable climatic conditions occurred at ∼6.5 Ma. Thus a significant decrease in the EA summer monsoon strength from 7–5.7 Ma in the Baode region, based on three samples dated at 7.0, 6.5, and 5.7 Ma [70], does not contradict the mollusk results. We suggest that much more definitive results would have been obtained if a higher sampling density had been used. In addition, numerous paleoclimatologists regard the occurrence of Red Clay sequences as reflecting aridity or desertification and an intensified winter monsoon [16], [37], [42], [43], [69], [71], which contradicts the mammal and isotope record.

We suggest four possible reasons for the discrepancies amongst the extant research results. First, the sensitivity of the proxies used may be one of the main reasons; and indeed, it has long been found in Quaternary loess studies that different climatic proxies can respond differently to climatic changes [55]. Second, as summarized by Wang et al. (2005) [72] and Kaakinen et al. (2006) [24], interpretations of expansions of C4 vegetation are contentious in terms of whether or not they are a summer monsoon proxy [2], [4], [45]. Third, regional difference in patterns of climate change may have occurred, as suggested by Passy et al. (2009) [27]. Finally, low stratigraphic resolution may have resulted in the failure to resolve environmental changes in sufficient detail, and high resolution mammalian faunal studies (for example using a 20-cm interval) may shed light on the observed differences. In summary, the conflicts cannot be resolved by the present study and future studies employing more sensitive proxies, higher resolution or continuous sampling combined with accurate dating may be necessary.

Second, from 6.2 to 5.4 Ma (Zone 2), the dominant CA terrestrial mollusks indicate that the climate during this interval remained cold and dry, but was not drier than in Zone 1, as evidenced by the slightly greater abundance of TH species and individuals. At the beginning of this zone, from about 6.2 to 5.8 Ma, the number of TH mollusk individuals was higher, and this corresponds to a declining rate of dust deposition as indicated by the thinness of the loess layers in the Dongwan sequence (Figure 4). This supports the finding that decreasing or increasing dust deposition affects the development of terrestrial mollusks by creating either more- or less- favorable environmental conditions [65]. After about 5.8 Ma the number of TH mollusk individuals was reduced, which is probably related to the increase of dust deposition in the CLP, as shown by the increased thickness of the loess represented by this interval (Figure 4). These cold, dry climatic conditions were not solely restricted to the western CLP; in the eastern CLP, mollusks in the Xifeng Red Clay sequence exhibit dominant percentages of the CA group associated with a few meso-xerophilous components and a lesser occurrence of TH species, also indicating a cold, dry climate [46]. This is also reflected by the coarse grain size of the Red Clay sequence in the CLP [16], and by the fact that dust deposition rates in the western Pacific remained high, albeit lower than before, and with a declining trend towards the subsequent time interval from 5.4 and 4.4 Ma [69]. The thickness of the Dongwan loess layers was not higher than before, except at about 6.2 and 5.6 Ma; however, the thickness of the soil layers does not change significantly. The δ^13^C values of the soil carbonates in the Lingtai and Xifeng Red Clay sequences were similar to those of the previous time interval from 7.1 to 6.2 Ma, indicating again that C4 plants were not the dominant vegetation in the studied area of the CLP [6], [9]. These lines of evidence indicate generally cold, dry climatic conditions in the EA continent but which were not drier than the lower stage. However, the global mean benthic foraminiferal δ^18^O record demonstrates that global ice volume was increasing with the highest values occurring at about 5.8–5.7 Ma, followed by a decrease towards the upper stage [1].

There is much evidence supporting the conclusion from the Dongwan mollusk results that the winter monsoon may still have been strong from 6.2–5.4 Ma but that the summer monsoon strengthens than before although may be to a lesser extent, as confirmed by the pedostratigraphy and iron geochemistry of the Lingtai Red Clay deposits in the CLP [20], [21]. The coeval pollen record from the Lingtai section exhibits a predominance of Chenopodiaceae and *Artemisia*, indicating a desert or desert-grassland landscape during the Late Miocene [73]. A higher dust accumulation rate and coarser eolian grain size in the CLP and in the North Pacific suggest stronger continental aridity in the Asian interior from ∼6.2 to ∼5 Ma [14], [16], [69]. The variation in the U-ratio of the grain size of the Red Clay, reflecting the changing strength of the winter monsoon, indicates a strong winter monsoon between 6.1 and 5.4 Ma [13]. The sedimentation rate across the CLP, including at the Baode Red Clay sequence, indicates that the EA winter monsoon strengthened between 6.26 and 5.4–5.25 Ma [14], [15].

Third, from 5.4 to 4.4 Ma (Zone 3), the prominent feature is that all of the CA species and individuals are very few in number and in fact they decrease significantly with almost no occurrence after about 5 Ma. In contrast, there are high values of the TH species numbers and individuals with the maxima occurring at around 5–5.1 Ma when a thick soil layer formed, indicating warm, moist climatic conditions. In the eastern CLP, the mollusk fauna was characterized by maximum abundance of TH species, an absence of CA species, and relatively abundant meso-xerophilous taxa [46]. The Xifeng pollen record indicates a significant increase of temperate forest plants, also implying humid regional climatic conditions during this period [74]. The δ^13^C values indicate a slight decrease and thus a shift towards more C3 plants in the CLP in agreement with the pollen results (Figure 4). Ding et al. (1999) identify extremely mature soils in the 5.5–3.85 Ma interval at Lingtai, and interpret these as indicating warm and wet climates, and possibly the strongest summer monsoons in the past 7 Ma [20]. Minimum grain size and sedimentation rate in the CLP [15], [16], decreased thickness of loess layers in the Dongwan sequence (Figure 4), and very low dust deposition rates in the western Pacific [69] together suggest very warm, moist climatic conditions in the CLP and less dry conditions in the Asian interior, the potential source area of dust in the CLP and western Pacific. However, this warm climate is not clearly recorded by the global mean benthic foraminiferal δ^18^O record compiled by Zachos et al. (2001) [1].

Last, from 4.4 to 3.5 Ma (Zone 4), all of the CA indices increase significantly and remain almost unchanged, while in contrast all of the TH indices exhibit a different pattern of variability with high values from 4.4 to 4.0 Ma, coincident with changes in the CA indices, an abrupt decrease from 4.0 Ma and increased values again from 3.7 Ma. The CA species during this time interval were different from those between 7.1 and 5.4 Ma. Indeed, the CA species of *Cathaica pulveraticula*, *Cathaica schensiensis*, and *Pupopsis retrodens* have only been identified in this zone, as shown by Li et al. (2006a) [58], probably indicating that climatic conditions became drier. The coeval Xifeng land snail record from the eastern CLP is dominated by meso-xerophilous taxa, associated with a significantly reduced abundance of TH species and the paucity of CA taxa during the late period [46]. Pollen data from the Xifeng Red Clay sequence indicate a typical steppe ecosystem during this period [74]. These variations are in good agreement with the expansion of C4 plants in the central CLP as observed at the Lingtai and Xifeng Red Clay sequences [6], [9]. In addition, the loess layers in the Dongwan section during this time interval are of moderate thickness, thinner than from 7.1 to 6.2 Ma but thicker than from 5.4 to 4.4 Ma. In contrast the paleosols are somewhat thicker than before (Figure 4), indicating longer pedogenesis under different climate conditions, and probably enhanced seasonality, corresponding to expansions of C4 plants in the central CLP [6], [9]. Pedostratigraphy and iron geochemistry of the Lingtai Red Clay deposits in the CLP indicate that the EA summer monsoon weakened significantly over the interval 3.85 to 3.15 Ma [20], [21]. Dust deposition increased again in the Pacific in parallel with increased aridity in the dust source areas [69] (Figure 4).

Moreover, the entire Dongwan terrestrial mollusk record provides information about the evolution of mollusk diversity and climatic changes from the Late Miocene to the Pliocene. Previous studies indicate that in most Quaternary loess sequences in Europe and North America generally fewer than 20 mollusk species can be identified and diversity varies with *H* values between 0 and 4, suggesting that the loess deposits provided few additional ecological niches for land snails to grow and develop [65]–[67]. In the Neogene Dongwan loess sequence in the CLP, East Asia, 24 mollusk species were identified and diversity varies between 0 and 2.5, which also suggests that fewer niches were provided. The response of the Neogene terrestrial mollusk populations to climatic changes depends on their ecological requirements. Different ecological groups, such as CA and TH, respond differently. During the time interval from 7.1 to 5.4 Ma when a cold, dry climate obtained, as indicated by a high flux of dust in the CLP and western Pacific, the diversity of the CA group was high, indicating that relatively cold, dry climatic conditions may be favorable for the development of CA terrestrial mollusk populations. In contrast, during the relatively warm, moist time interval from 5.4 to 4.4 Ma, corresponding to a reduced loess thickness in the CLP and low dust flux in the western Pacific, the diversity of the TH terrestrial mollusk populations was high.

### Possible Causes of Paleoecological and Paleoclimatic Evolution in the Western CLP during the Late Miocene and Pliocene

Dust in the Asian interior, including northwestern China, was emitted and transported in two possible modes, corresponding to patterns of low and high level atmospheric circulation. One mode is that the Asian dust was transported eastwards by high atmospheric circulations (westerlies) and reached northwestern Pacific, as recorded by high values of dust flux to the northwestern Pacific. The other mode is that dust in the Asia interior was transported by low level atmospheric circulation, the EA winter monsoon, to the middle reaches of the Yellow River, leading to the formation of the CLP [37], [49], [75], [76]. Thus loess sequences in the CLP and dust deposits in the western Pacific both relate to climatic changes that impacted the regions to the north of the Tibetan Plateau, i.e., the Asian interior, as has been indicated by the previous studies of Hovan et al., (1989) [77] and Rea et al., (1998) [69].

At approximately 8–7 Ma, the accumulation rate of eolian deposits in the CLP reached high values of about 4 cm/ka [37] and dust deposition in the western Pacific was maximal [69] (Figure 4), indicating that particularly dry climatic conditions must have prevailed in the Asian interior resulting in the mobilization and transport of large amounts of dust. Indeed, a palynological study of the Late Miocene–Pliocene sediments of the Dushanzi section from northwestern China (Figure 1) indicates that steppe taxa (*Artemisia* and Chenopodiaceae) were generally dominant, implying that a dry climate existed in the inland basins of northwestern China since 8.7 Ma, except for a warm and humid phase that lasted from 5.8 to 3.9 Ma [78]. In addition, significant desertification in northwestern China prevailed as early as 7.2–7 Ma, as shown by the development of eolian sand dunes in the Taklimakan Desert [79] and eolian Red Clay deposition around the Lanzhou region [80]. These climatic and environmental conditions probably provided a suitable environment for the growth and development of C4 plants in northern China and promoted the expansion of eolian deposits from the west towards the eastern part of the CLP [42], [43] and which constituted suitable environments for terrestrial mollusks. Thus terrestrial mollusks grew and developed in the CLP and were able to record the patterns of environmental evolution as recorded by the Dongwan sequence.

Variations in the Dongwan terrestrial mollusk assemblages indicate that, during the time interval from 7.1 to 3.5 Ma, major paleoecological and paleoclimatic changes occurred at about 6.2, 5.4, and 4.4 Ma. The change at around 6.2 Ma, the boundary between Zones 1 and 2, is less significant than the one at 4.4 Ma, and therefore it is not clearly reflected in terms of a transition in periodicity recorded by the relative abundance of mollusks at this time [47]. Indeed, a relatively high number of CA mollusk species and individuals, consistent with the high relative abundance of CA mollusks [47], prevailed during Zone 1 and Zone 2, indicating that a cold, arid climate prevailed in the study area. These two zones exhibit the same dominant 100 kyr periodicity [47], again suggesting that the 6.2 Ma datum was not particularly significant at Dongwan. However, the TH mollusk individuals and species are somewhat different within these two zones, and in addition the appearance of mollusks in the Xifeng Red Clay sequence and the upper age of the QA-I section imply that 6.2 Ma may be an important datum in the paleoclimatic evolution of the CLP [37], [46].

Changes in the CA mollusks of Zones 1 and 2 are paralleled by a global cooling trend [1] (Figure 4), increased ice-rafted detrital flux in the Northern Hemisphere [81]–[85], and the buildup of the Western Antarctic ice sheet [1]. The CLP is particularly sensitive to changes in high northern latitudes through the EA winter monsoon circulation [76], [86]. Extended ice sheets in the Northern Hemisphere reinforce the southward movement of cold air and thereby enhance the Siberian High that controls the EA winter monsoon wind system [86]. Thus, global cooling, especially the expansion of ice deposits in high northern latitudes, could have affected the Siberian High and the EA winter monsoon, thereby expanding habitats for the CA mollusks in the CLP.

There is no evidence for major uplift of the Tibetan Plateau at about 6.2 Ma, and therefore this mechanism is excluded as a cause of the change at this time. However, if the Tibetan Plateau reached a significant height at about 8 Ma it would have accelerated climatic cooling and strengthened the EA winter monsoon [3], [8], [87]–[90], causing cold, dry climatic conditions in the western CLP during the Late Miocene, as discussed above.

The shift at about 5.3–5.4 Ma roughly corresponds to the onset of the Pliocene when global climate became warmer than before. However, the climatic drivers contributing to the Pliocene global warming are still highly debated. As summarized by Haywood et al. (2009) [91], possible candidates include paleogeographic changes [92], altered atmospheric trace gas concentrations and water vapor content [93], changes in oceanic circulation [94], [95], oceanic heat transport [5], [96], thermal structure of the oceans [97]–[99], and feedbacks generated through altered land cover (including ice sheet extent), surface albedo, cloud cover and temperature [100].

Our results seem to support the suggestion that the warming interval may be related to changes in ocean circulation and ocean heat transport caused by the closures of the Panama and Indonesian seaways [5], [94]–[96]. Closure of these two seaways may have caused changes in heat distribution between the Pacific and Atlantic, causing reorganization of global climatic patterns and changing the pattern of atmospheric moisture flux from latitudinal to meridional, resulting in increased moisture flux to high latitudes [5], [95], [101] and contributing to climate changes in the CLP. Moreover, both geological records and modeling studies show that closures of the Panama and Indonesian seaways likely played important roles in the strengthening and enlargement of the western Pacific warm pool [5], [102]–[106], providing more moisture and heat to the CLP favorable for the abundant occurrence of TH mollusk species.

The 4.4 Ma shift, representing a climatic transition from early Pliocene warming to late Pliocene cooling, is observed in the Dongwan mollusk diversity and species numbers and individuals; however, it is not clearly shown in the relative abundance of mollusks [47]. It may be related to tectonic events such as the uplift of Tibetan Plateau, since climate models suggest that uplift played a particularly important role in the evolution of the global paleoenvironment [8], [87]–[90], and the effects of which on the EA winter monsoon are more significant than on the summer monsoon [89]. Changes in depositional facies from distal alluvial plains to proximal alluvial fans and an increase in sedimentation rate near Yecheng (Figure 1) in the western Kunlun Mountains indicate the uplift of the northern Tibetan Plateau at about 4.5 Ma [7]. This tectonic activity is thought to trigger not only the enhancement of the EA monsoon, but is also considered to be a driver of general cooling through the consequent increase in the rate of chemical weathering, thus accelerating ice expansion in Northern Hemisphere high latitudes [88], [107], [108] and further strengthening the Siberian High and the EA winter monsoon and transportation of dust to the CLP. Furthermore, uplift of the Tibetan Plateau could have blocked moisture transport to the Asian interior, contributing to its aridification. Under these climatic conditions, the northern CLP would have commenced a drying trend earlier than the southern CLP, causing significant ecological changes in terrestrial ecosystems including terrestrial mollusks and C4 plants at roughly 4.4 Ma.

## Conclusions

The Dongwan terrestrial mollusk record from the western CLP exhibits four stages during the time interval from 7.1 to 3.5 Ma, indicating the phased evolution of paleoecology and paleoclimate. From 7.1 to 6.2 Ma, very cold and dry climatic conditions prevailed. From 6.2 to 5.4 Ma, the climate remained cold and dry but was not as dry as the preceding interval, as evidenced by the dominance of CA mollusks and rather more TH species and individuals. From 5.4 to 4.4 Ma, very warm and moist climatic conditions prevailed, evidenced by high values of the TH species and individuals, as well as by the very small numbers of CA species and individuals and their almost complete absence after about 5 Ma. From 4.4 to 3.5 Ma, all of the CA indices increase significantly and remain at a high level, indicating a cooling climate; however, all of the TH indices exhibit relatively high valuesfrom 4.4 to 4.0 Ma, an abrupt decrease from 4.0 Ma and an increase again from 3.7 Ma. The three CA species, *Cathaica pulveraticula*, *Cathaica schensiensis*, *Pupopsis retrodens*, occurred solely during this period and were absent from 7.1 to 5.4 Ma, suggesting that the climate from 4.4 to 3.5 Ma was becoming colder and drier than previous stage.

The very cold and arid climatic conditions, with changes at about 6.2 Ma, are paralleled by a global cooling trend, increased in ice-rafted detrital flux in the Northern Hemisphere [81]–[85], and the buildup of the Western Antarctica ice sheet [1]. The shift at about 5.3–5.4 Ma roughly corresponds to the onset of the Pliocene when global climate became increasingly warmer than previously. Our results seem to support the suggestion that the warming interval may be related to changes in oceanic circulation and oceanic heat transport [5], [94]–[96]. The 4.4 Ma shift may be related to tectonic events such as the uplift of Tibetan Plateau.

Variations in the diversity of the CA and TH mollusks, *H*(CA) and *H*(TH), are closely related to climatic changes during the Late Miocene to Pliocene. From 7.1 to 5.4 Ma when a cold, dry climate prevailed, the CA group was the more diverse. In contrast, during the relatively warm, wet time interval from 5.4 to 4.4 Ma, the TH terrestrial mollusk populations became more diverse. It should be pointed out that most of the Neogene terrestrial mollusks in the CLP have modern representatives and therefore they have the potential to estimate quantitatively Neogene changes in temperature and precipitation in the CLP. However, such estimates depend upon the development of a training set based on a large number of surface samples. Changes in fossil terrestrial mollusk diversity are significant for the prediction of terrestrial biodiversity changes, and Quaternary loess deposits in Europe and North America have been studied in this context. The 22-Ma loess deposits in China provide an excellent opportunity for understanding long term changes in terrestrial mollusk diversity; and although the present paper focuses on the Late Neogene terrestrial mollusk in the CLP, ongoing studies will focus on other time intervals.

## Supporting Information

Table S1 **The depth, age, and mollusk data from the Dongwan loess-paleosol sequence**. S(CA)–Total number of species of the cold-aridiphilous (CA) mollusk group. *H*(CA)–Diversity of the CA mollusk group. CAMI–Mollusk individuals of the CA mollusk group. S(TH)–Total number of species of the thermo-humidiphilous (TH) mollusk group. *H*(TH)–Diversity of the TH mollusk group. THMI–Mollusk individuals of the TH mollusk group. S(total)–Total number of species of the total mollusk group. *H*(total)–Diversity of the total mollusk group. E(total)–Equitability of the total mollusk group. TMI–Total Mollusk individuals.
(XLS)
